# Maritime Environment Assessment and Management Using through Balanced Scorecard by Using DEMATEL and ANP Technique

**DOI:** 10.3390/ijerph19052873

**Published:** 2022-03-01

**Authors:** Wen Cheng Lin

**Affiliations:** Department of Business Administration, National Taipei University of Business, Taipei 10051, Taiwan; wencheng@ntub.edu.tw

**Keywords:** maritime environment analysis, balanced scorecard, DEMATEL, ANP

## Abstract

Previous studies have found that the occurrence of maritime accidents often lacks a sound environment management mechanism. The reason is that maritime safety needs management functions to promote each other. This study aims to assess the risk analysis of maritime accidents, applying balanced scorecard (BSC) concepts integrating decision-making trial and evaluation laboratory (DEMATEL) with analytic network process (ANP). The empirical results are that the balanced scorecard could be applied as a maritime procedure management method in maritime risk analysis. A total of 30 questionnaires were collected via scholar questionnaire, and five criteria or key factors for strengthening risk assessment of marine accidents were determined. According to the application of BSC, the risk analysis criteria constructed can assist maritime authorities to reduce the maritime accidents.

## 1. Introduction

For an island nation, it is very important to ensure the safety of coastal shipping around the port. Safety navigation could reduce costs for maritime incidents and pollution and increase harbor productivity and competitiveness. In order to avoid maritime accidents, the United Nations promulgated the Convention on the Law of the Sea in 1969 and 1982. The Convention covers the disposal of oil pollution accidents on the high seas, which clearly defined the meaning of maritime casualties and marine accidents. Resolution Maritime Safety Committee (MSC). 255 (84) of the International Maritime Organization clearly stipulates the types of maritime accidents and promulgates the code of international standards and recommended practices for safety investigation of maritime casualties or maritime accidents [1].

The evaluation of the shipping industry clearly shows that human factors are still the main cause of maritime accidents. Recently, the international maritime authority has made great contributions to improving the maritime safety of the shipping industry. However, the total number of ship accidents has not decreased significantly [2]. Although working in the marine has a good salary, it has been exposed to a high-risk environment for a long time. The negative effects of maritime accidents can be summarized as loss of lives at sea; environmental pollution, mainly due to oil spills; and economic results that may deteriorate the international logistic flow [3]. It has been very well-documented that the main reason for a maritime accident is the “human factor”. No matter what position, working as a docker in the port, a sailor on board, or working in any position at sea, these jobs require the payment of manual labor, and need to work at sea for a long time, which will increase the risk of accidents for seafarers. The International Maritime Organization (IMO) requires Member States to conduct a safety investigation in case of marine accidents. From the review of relevant literature and survey results, it is found that maritime investigations often investigate the causes of accidents after they occur and often lack a set of risk management mechanisms. The related work pointed out that 75% to 96% of maritime causalities are linked to human error engaged with professional maritime transportation [4,5,6,7]. As reported by [8], human error contributes to 89–96% of collisions, 75% of fires and explosions, 79% of towing vessel groundings, 84–88% of tanker accidents, and 75% of allusions.

The maritime operating environment is undergoing major transformations, such as changing demand patterns, intensifying global competition, and adecline in funding. Therefore, maritime education institutes need a careful evaluation of the human factors that influence students’ selection of higher education institutes. Maritime education must be based on human factor training [9]. However, the balanced scorecard is a strategic management planning system, which is widely used in business management and non-profit or government organizations. It puts forward the vision and strategy in line with the organization and achieves the organization’s financial performance through internal process improvement and external communication. The maritime industry also needs a balanced scorecard concept to manage or process maritime accidents. In the past, few papers have linked maritime safety and the balanced scorecard together; the balanced scorecard is more than just a measurement system. Multiple criteria decision making (MCDM) is a branch of operational research dealing with finding optimal results in complex scenarios including various indicators, conflicting objectives, and criteria. This tool is becoming popular in the field of energy planning due to the flexibility it provides to the decision makers to make decisions while considering all the criteria and objectives simultaneously. It enables organizations to articulate their vision and translate strategy into action. It provides a cycle of internal processes and external communication, with continuous improvement as strategic performance and results. This paper aims to achieve these objectives (1) to examine the environment factors on human factors that contribute to the maritime accident applying BSC and (2) to prioritize the significant factors with the maritime safety criteria through fuzzy AHP.

The main contribution of this study is the combination of a multi-criteria method and a quantitative risk analysis technique. This paper consists of five parts: (1) the introduction; (2) the literature review, which lists the latest research results in the field of multi-standard methods and the balanced scorecard; (3) the part that puts forward the concept of a fuzzy extended analytic hierarchy process; (4) the results; and (5) the conclusion.

## 2. Literature Review

### 2.1. Maritime Accident and Education

Maritime accidents are often caused by human factors. Hansen mentioned that the human factor is defined as a result or effect of human actions, the causal factor of an accident, deliberate violation, and the actual action taken by a human being [10]. In general, researchers rarely agree on either a specific definition or how to prevent human factors. The concept of environment factor is examined as currently used in the literature of a variety of industries and professions. As for this research, the definition of the environment factor is absolutely focused on the maritime industry. Hansen et al. illustrated 1993 maritime accidents among crew aboard Danish merchant ships in the period 1993–1997; this study found that demographic and related word variables impact safety outcomes [11]. Ships operate in a highly risky milieu; typically, the people on board adapt a set routine of shift work disrupted by arrival at, working in, and sailing from ports. Recently, international maritime authorities have performed significant contributions to improve safety at sea in the shipping transportation industry. Inadequate safety of ship operations is not only caused by the material precautions but also human factors such as seafarers’ behaviors, habits, lack of attention, and occupational educations [12]. Most marine accidents are caused by some form of human error as well as incidents [13,14].

Therefore, the study of human factors and accident analysis has become a popular research topic for maritime professionals. Within this framework, the whole maritime system is a man-made system, which depends on real professionals, but the center of the problem is the “man” himself [15]. In fact, there are many studies on the role of the human factor in maritime accidents and incidents [4,5,6,7,8,16]. Furthermore, many authorities such as “The Australian Transport Safety Bureau”, “Transport Safety Board of Canada”, “The Maritime Safety Authority of New Zealand”, “The Marine Accident Investigation Branch of United Kingdom”, or “The National Transportation Safety Board of USA” reported via internet that human error is the most important factor in maritime accidents.

Human factors start from maritime education and training. Human factors can be defined as people who are endowed with a series of abilities, talents, and attitudes in an organization [17]. In order to meet the needs of the industry, the primary task of maritime education and training institutions is to educate and train seafarers with comprehensive knowledge and skills. Recruiting sufficiently qualified and experienced staff in education institutions is a large challenge. Froholdt and Hansen pointed out that there are many incompetent teachers in training and education institutions. In some cases, the teachers employed have no teaching ability, which is worse; the worse teachers are assigned teaching roles. Therefore, it is a challenge for the academic community and researchers to determine which cadre can become a better teacher. Maritime Education and seafarers’ ability training are important parts of education and training institutions and human resource development strategies [18]. Therefore, determination of maritime accidents caused by human errors is a kind of multiple criteria decision-making (MCDM) problem and requires MCDM methods to solve it. Although the human error on maritime accidents is a kind of MCDM problem, there are limited MCDM studies that focus on identifying the main causes of human errors in maritime accidents. Consequently, a hybrid MCDM method can be followed to handle the problem appropriately [13,19].

### 2.2. Balanced Scorecard

BSC concepts consider the strategy and vision of organization and focus on nonfinancial and financial performance. In other words, it illustrates short-term and long-term financial performance, also emphasizing competency advantage [20,21,22]. An entire BSC evaluated performance from four perspectives [20]: financial, customer, internal business, and innovation and learning.

The financial perspective can capture the cash flow, sales growth, and profitability of enterprises; the goal of the customer perspective is to provide customers with higher customer value; the internal process perspective focuses on the smoothness of the internal operation process; the learning and growth perspective promotes organizational innovation by strengthening human resources and information technology.

This paper applies the four perspectives of BSC to the process of maritime assessment. Maritime safety risk should also be studied and developed from the perspective of the balanced scorecard. The reconstruction of maritime education can improve the learning level of seafarers and attach importance to maritime safety. The performance basis of seafarers can be evaluated through the case materials of maritime accidents. Like the balanced scorecard, through the good education and growth of employees, the organization can refine the internal process, such as the training management of port or ship crew. Maritime seafarers can play the role of maritime inspectors. According to the balanced scorecard, internal processes are the antecedents of customer satisfaction. If seafarers can berth smoothly at sea and reduce the occurrence of maritime accidents, it is just like the balanced scorecard with customer satisfaction as the goal. The purpose of a skilled crew operation is to reduce the probability of marine accidents. Finally, the goal of the balanced scorecard is to improve the financial performance of enterprises, and maritime safety reporting is just like the financial performance of the balanced scorecard. The maritime safety report records the details of maritime accidents. It can review, track, and revise the current situation of reducing the risk of maritime accidents.

In this study, we used a quantitative application to examine the reasons for work-related accidents in ships and tried to discover alternative solutions for the matter. For the solutions of such problems in which various factors and criteria must be analyzed and evaluated, using a DEMATEL with ANP can be a positive approach. In this study, we determined the criteria that cause work-related accidents and offered an appropriate methodology for the solution of the problem by using a MCDM model. ANP is used to determine and rank the accident-related criteria according to their importance.

## 3. Methodology

DEMATEL and ANP are proposed by a hybrid MCDM model to determine the impact of each perspective and criterion and measure the importance of each factor.

### 3.1. Data Collection

**Figure 1:**
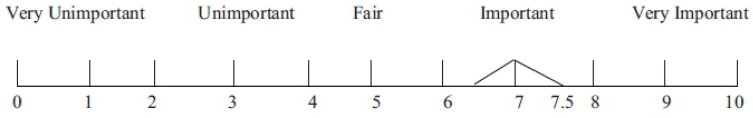
Rank of importance level.

From the maritime assessment factors in Table 1, a series of factors that can be used to assess marine accidents are collected. The purpose of the questionnaire is to include these. In the first stage, the relative importance criteria (average 7.5 and above) were selected by asking experts to answer the questionnaire. The respondents answer ranges from 0 to 10, with a high score indicating that it is very important, as shown in Figure 1. In this study, three maritime scholars, six shipowners, and one government official in charge of maritime affairs were asked to fill in the answer. Ten respondents in areas of expertise adjusted and gave comments on the preset perspectives and indicators of this study and, finally, sorted out maritime incident assessment factors. In the second stage, the important scale based on triangular fuzzy number (average value 7.5 and above) was established.

In the second stage, using the results of the first stage, the method of ANP and DEMATEL was combined into the questionnaire design. The questionnaire obtained the order of importance of comparing the paired results. The focus of the investigation was on the relevant factors of marine accidents. Through interviews and completed surveys, the respondents’ opinions and thoughts on the evaluation criteria were collected. In total, 30 questionnaires were collected from June 2020 to July 2020. The 30 respondents came from maritime investigators of Taiwan Transportation Safety Board and Maritime Port Bureau. They not only passed the examination for public servants but also had practical experience in the maritime industry for more than 10 years. Each interview with the interviewees took 30–40 min. They were collected via the interview model.

### 3.2. DEMATEL

The DEMATEL approach was devised by Geneva between 1972 and 1976 to solve unclear issues and complicated problems [23]. This technique is used to solve complex and dependent problem groups [24]. In particular, this method has been adopted by different researchers to analyze intertwined group among criteria in multicriteria decision-making. In addition, they stressed that DEMATEL is generally considered to be one of the best functional technologies for finding causal relationships between evaluation criteria in the evaluation process of any system or product [24]. Another advantage identified by [25] is that, when the DEMATEL method is used, the number of evaluation criteria selected will be reduced, which will help organizations improve the efficiency of specific factors based on the effect-oriented graph. In fact, due to the imperfection of some evaluation criteria and even the existence of uncertain factors, human judgment is basically fuzzy, and it is difficult to evaluate with accurate brittle value [26]. This is why fuzzy theory is used in the DEMATEL method to overcome this kind of MCDM problem. The fuzzy DEMATEL method is applied to different research fields to solve different MCDM problems [24,27,28].

Step 1: Define the decision problem and evaluation criteria: in this step, a critical review of the literature is required to explore and collect relevant data. Expert judgment is crucial at this point, which is why it is necessary to establish an expert decision-making group to discuss this issue in order to achieve this goal. According to the collected expert information opinions, the possible factors were selected and finally determined as the evaluation criteria.

Step 2: Formulate the average and initial relation matrix (a): the average matrix is composed of the beginning relation matrix. The beginning relation matrix is based on the direct influence between any two factors and is determined by experts. The experts are required to grade the factors on the given scale: “no influence: denote 0”; “small influence: denote 1”; “high influence: denote 2”; “very high influence: denote 3”.

The x_ij_ representation specifies the influence of expert’s opinion on criteria i on criteria j. When i = j, the value of the cell is set to 0, which means there is no impact. For each respondent, (n × n) nonnegative matrix can be designed as X_k_ = [xk_ij_], where ‘k’ is the number of respondents (1 ≤ k ≤ H); n is the number of factors. Thus, X^1^, X^2^, X^3^…, X^H^ is the matrices from H respondents. In order to synthesize all the opinions of H experts, the average matrix A = [a_ij_] can be constructed as follows:

(1) $$a_{\mathrm{ij}}=\frac{1}{H}\sum_{K=1}^{H}x_{\mathrm{ij}}^{k}$$

Step 3: Calculation of normalized initial direct relation matrix (D): can be performed using the formula,

(2) D=A×S

where, S = min$\left[\frac{1}{max\sum_{j=1}^{n}\left|a_{ij}\right|},\frac{1}{max\sum_{i=1}^{n}\left|a_{ij}\right|}\right]$ Every component of matrix D is bounded between 0–1.

Step 4: Find the total relation matrix (T): the total relation matrix can be defined by Step 3.

(3) $$T=D{\left(I-D\right)}^{-1}$$

The sum of the rows and columns of the total relation matrix T are calculated. If r_i_ is the sum of row ith in the matrix T, then r_i_ summarizes the indirect and direct effects of factor i on other factors. If c_j_ is the sum of column jth in the matrix T, then c_j_ represents the indirect and direct influences that factor j receives from other factors. Additionally, (r_i_ + c_j_) are called “Prominence” and represent the total effect of factor i given and received. (r_i_ + c_j_) represents the importance of criteria i in the whole process. In contrast, the difference (r_i_ − c_j_) is called “Relation”, which denotes the net effect of factor i on the system. Specifically, if (r_i_ − c_j)_ is positive, then factor i is the cause factor. If (r_i_ − c_j_) is negative, the coefficient i is a receiver factor [25].

Step 5: Set threshold: the average value of the components in the total relation matrix T attains threshold value. Because matrix T provides an example of how one criterion affects another, in this case, thresholds help to filter out insignificant or negligible effects. In addition, effects larger than the threshold value will be selected and represented in a directed graph. By mapping the data set of (r + c, r − c), we can obtain the directed graph.

### 3.3. Combining DEMATEL with ANP to Find the Important Weights

In determining the relative weights of these maritime analysis, several evaluation criteria are usually considered. These factors are usually difficult to obtain and interdependent of the weights’ relationship. DEMATEL technology is not used to determine the factors influencing the interaction, but to obtain accurate weights. This paper uses the ANP tool for discovering interactions. In short, ANP does not require a strict hierarchy, so it can include single or multiple networks. The ANP technique has been successfully used for many practical complicated decision problems, such as enterprise risk management, hot spring hotel service quality evaluation, and innovative cooking development [28,29,30,31,32]. It provides a method of input judgment and measurement to derive the ratio proportion priority of influence distribution between criteria and groups in decision-making procedure. Since the procedure is based on ratio metrics, resources could be allocated according to the priority of the ratio metrics.

Since performance criteria are usually interactive, direct and indirect influences are the key factors to evaluate performance. This paper uses DANP to evaluate the performance accurately. Saaty proposed a method to analyze ANP, which adopted the limit process method of the super-matrix [33]. ANP can be used to deal with interdependencies in theory; it is wise to use DEMATEL technology to generate causality in the first place.

## 4. Results and Discussion

This part includes the relationship between the analysis of maritime administrative performance and the measurement of performance criterion. According to the interviews/questionnaires filled out by the maritime administration owners, we find the key standards to illustrate the performance evaluation model.

### 4.1. Background and Problem Description

The coastline in Taiwan is 1140 km long, separated from Eurasia by the Pacific Ocean. Therefore, Taiwan has a superior transportation position in Asia. At present, over 90% of Taiwan’s resources are transported by sea. Taiwan ranks 14th among the top 20 container ports in the world. The rapid development of China’s economy has promoted the growth of economy, trade, and shipping between Taiwan and the Chinese mainland; an increase in the number of maritime ships in Taiwan and other countries was also observed. This information shows that Taiwan plays a key maritime role in Asia and the world. However, it means that any maritime accident in Taiwan will have a negative impact on the maritime transportation of Taiwan, China, Asia, and even the world.

**Figure 2:**
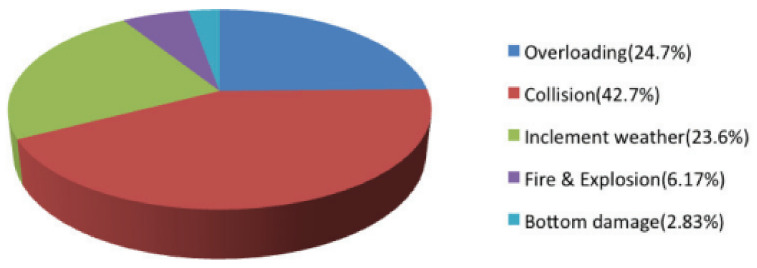
Causes of maritime accidents in Taiwan of 2013–2018 [34].

A total of 547 maritime accidents were recorded in Taiwan from 2013 to 2018 [34]. Figure 2 shows the principal causes of maritime accidents. In terms of numbers, collision (42.7%) and overloading (24.7%) accounted for 67.4% of the total accidents, and inclement weather accounted for 23.6% of the total accidents. The remaining marine accidents accounted for less than 9%, which pertained to leaking and extreme misfortune. These statistics reflect that hull/machinery damage, grounding/stranding, and fire/explosion did not change significantly in recent years, but collision/contact and others seemed to increase. Moreover, the location of marine accidents can serve as a basis for marine treatment.

### 4.2. Analysis of Results

According to DEMATEL formulation, respondents used scales of 0, 1, 2, 3, and 4 to indicate the degree of direct influence: “no influence”, “low influence”, “medium influence”, “high influence”, and “very high influence”, respectively. Table 2 shows the NRM of the relationship between the total influence matrix *T* and each perspective.

Some criteria have a positive d_i_ − r_i_, so they have a great influence on others. These criteria are referred to as dispatchers; other criteria have a negative d_i_ − r_i_ value and are greatly influenced by other criteria. These receivers are called receivers. The value of d_i_ + r_i_ represents the degree of relationship between each criterion and other criteria. The criterion with higher d_i_ + r_i_ value has stronger relationship with other criteria, while the criterion with lower d_i_ + r_i_ value has weaker relationship with other criteria. The significant positive d_i_ − r_i_ indicates that the influence of the criterion on other criteria is much greater than that of other criteria, which means that the criterion should be improved first. In the sense of management, the results of DEMATEL’s research can provide insights into how companies can improve their performance according to the criteria that have the greatest impact on other criteria.

As can be seen from Table 2, a (learning and growth) is the first in the intensity index of giving and receiving influence (5.765 in total sum (d_A_ + r_A_)); B (internal processes) is second; and C (seaworthiness) is the next. In short, learning and growth (A) is the most important influencing factor. The safety report (D) affects the other factors the least (3.315 in total sum (d_D_ + r_D_)). Due to improper operation or inadequate training of seafarers, risk assessment should focus on personnel learning and growth. Therefore, the learning and growth (A) perspective has the strongest relationships with the other perspectives. In addition, the values of d_i_ − r_i_ for the A, B, and C perspectives are positive, meaning that they affect other factors in the perspectives. If the D values of d_i_ − r_i_ are negative, it means that the criteria are influenced by other criteria.

**Figure 3:**
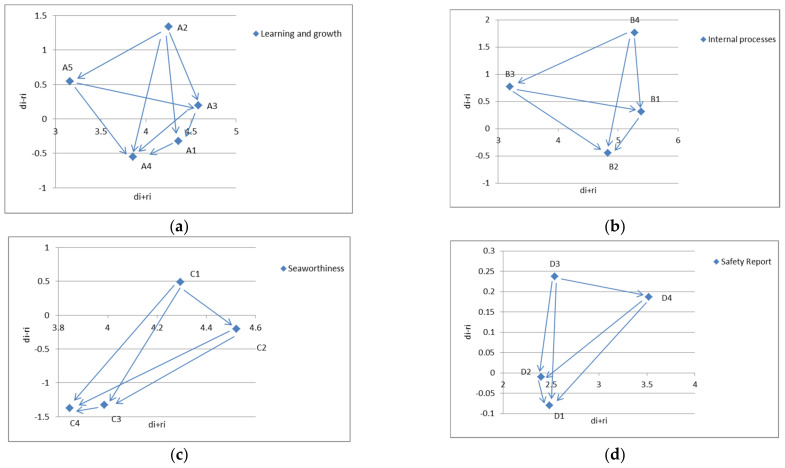
Causal diagram of total relationships. (**a**): Learning and growth criteria; (**b**): Internal processed criteria; (**c**): Seaworthiness criteria; (**d**): Safety Report criteria; d_i_—dispatchers; r_i_—relationships.

Table 3 shows the degree of influence of each criterion with the effect directly or indirectly. Ability to respond to emergencies in total sum (B1) is the most important consideration (d_B1_ + r_B1_ = 5.387); in short, the safety report (D2) is the criteria that has the least influence on other criteria (d_D2_ + r_D2_ = 2.398) in total sum. The d_b4_ − r_b4_ for the maximum accident information compilation (B4) indicates that it has the greatest direct influence on others (d_b4_ − r_b4_ = 1.762) in total difference; whereas cargo owner relationship management (C4) is the most influenced criterion by other criteria (d_C4_ − r_C4_ = −1.374) in total difference, as seen in Figure 3.

In addition, according to d_i_ + r_i_ and d_i_ − r_i_, the safety reporting criteria are the lowest of all other criteria. The results of this study are basically consistent with Kaplan and Norton (2001) regarding the strategy map and performance management used by the BSC concept. For example, the perspective of (A) learning and growth has an influence positively on the (B) internal process, (C) seaworthiness, and (D) safety report. The factor of internal process (B) has a positive influence on the factor of seaworthiness (C) and the safety report (D). Other results have shown that airworthiness (C) has an influence result on the safety report (D).

In this study, we use the methods of DEMATEL and ANP to obtain important indicators, combined with our survey data of marine relations personnel. The interrelationship between variables could be realized by illustrating their importance to the unweighted hypermatrix. By considering the influence of different perspectives, this paper prepared a weighted hypermatrix. The weights of various factors (global weights) are obtained by using the limits of the hypermatrix (Table 4). The ANP method derives the local weights, their respective hierarchies and global weights. It helps to realize the overall absolute weight of each criterion. Then, the attributes are obtained from their global weight. The empirical result is to identify the main criteria that maritime authorities will consider when seeking to improve risk factors. The results are shown in Table 4.

### 4.3. Discussion

Table 4 shows that, among the 17 criteria, maritime relation personnel believe that seafarer professional ability could be the first priority, with a weight of 0.087, followed by ability to respond to emergencies (0.078). The importance of the third to the tenth factors is in descending order: seafarer satisfaction (0.077), accident information compilation (0.076), seafarer education (0.074), marine management efficiency enhancement (0.069), seafarer resignation rate (0.068), market share (0.064), environmental hygiene and cleaning operation training (0.061), and cargo owner relationship management (0.060). Inside the top ten criteria, these are four dimensions of learning and growth, four from the dimension of internal process, two from the aspect of seaworthiness, and none from the perspective of the safety report. As shown in Table 4, the safety report is less important. The empirical results show that financial indicators are a final influence, which is consistent with the DEMATEL analysis.

In terms of performance standards, most scholars pointed out that, in most industries, there are three to six key factors leading to success [33]. The ANP is used as the top five risk factors in this paper. This study hoped that it can provide a reference for the risk control of the maritime industry and make it successful in the face of maritime accident emergencies and market changes. In order to strengthen the risk control of marine industry, the following suggestions are put forward.

#### 4.3.1. Seafarer Professional Ability

In the face of the recruitment crisis of navigation majors in higher vocational colleges, the investment in navigation majors in some traditional navigation colleges has declined, and the advantages of navigation majors have gradually weakened. In order to improve the professional competence of seafarers working on international merchant ships, shipping companies can use multi-standard methods to develop a conceptual framework for evaluating seafarers’ ability; they include three main standard groups (i.e., professional ability, interpersonal skills, and work attitude), determine the advantages and disadvantages of seafarers, and effectively point out the gap between the industry and seafarers’ own ability expectations. Strengthening the professional ability of navigation is ra equirement based on International Maritime Organization (IMO) standard course 7.03, so as to specifically improve the quality of students, meet the requirements of international conventions and norms, and achieve the professional ability of the personnel engaged in maritime industry after graduation [35,36].

#### 4.3.2. Ability to Respond to Emergencies

With the increasing automation of navigation equipment and the increasing connections between various parts of the system, the crew’s ability to judge various maritime hazards is also limited. To improve the ability to deal with maritime accidents, this study analyzed maritime accident cases to identify which competencies ships’ officers were lacking, compared the emergency response competencies required by international conventions, analyzed various emergency management manuals of shipping companies, and carried out questionnaire surveys to suggest improvements of emergency response capability for ship’s master. At this time, it is necessary to train some crew members to have a new safety concept of the new voyage instrument. Especially in the analysis and judgment of some emergencies, in order to successfully remove the accident crisis, it is necessary to have specific reference documents during navigation [35,37].

#### 4.3.3. Seafarer Satisfaction

In general, the interviewees reflected strongly on the problems such as on-board inspection, heavy workload, ship and shore management, and mental loneliness, especially loneliness and social loss far away from their families, which were the most influential factors on the unhappy of seafarers [38]. It is found that some seafarers are more and more dissatisfied with the ship’s environment. Job satisfaction is considerably correlated with job performance of seafarers. In addition, the amount of stress associated with working onboard a ship and attractiveness of rewards are key determinants of job satisfaction. The dispositions of seafarers and appeal of the job design also have considerable impacts on job satisfaction. Although they are impressed by all kinds of modern equipment of the new ship, these internal environments are often extremely demanding, which limits the range of crew activities and reduces the space for some crew members to interact with each other.

#### 4.3.4. Accident Information Compilation

In the case of ship collision or maritime accidents in the sea area of our country, in addition to the rescue of life and property safety at sea, the Maritime and Port Bureau (MPB) of the Ministry of Transportation and Communications should convene experts and scholars from the maritime sector to form a maritime assessment group to determine the responsibility of each party for the accident factors of the maritime accident. It is more important in the field of health and safety, especially in the maritime industry. Therefore, the shipping industry evaluates the importance of seafarers’ personality traits and workplace risk behavior. The specific method can determine which dimension of personality traits (five models) is more effective for occupational accidents, so as to propose a new model that helps to reduce the accident rate [38].

#### 4.3.5. Seafarer Education

Taiwan is surrounded by the sea, and land resources are scarce. The national economy and people’s livelihood depend on sea transportation. Therefore, the development of maritime transportation has become an important issue in Taiwan’s economic construction. Therefore, the strengthening and upgrading of maritime education is the main link to coordinate with the national economic construction [35,36].

**Figure 4:**
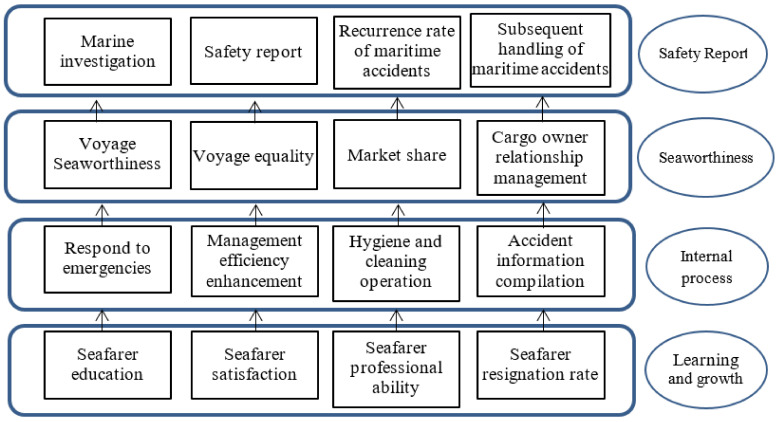
Risk accident assessment map.

According to the strategic map developed by [37], as shown in Figure 4, this paper illustrated a designed strategy map to enhance risk accident analysis. Like the BSC dimension, internal processes promote the customer satisfaction. Marine seaworthiness is the same as the objective of BSC customer satisfaction. The proficiency of crew operation is to reduce the probability of maritime accidents. Finally, the strategy map aims at the financial results of the enterprise, and the maritime safety report is just like the financial dimension of BSC. This result can be compared with the past literature and effectively apply strategic management to environmental risk assessment [35,36].

## 5. Conclusions and Remarks

This paper applied BSC to assess risk analysis and evaluation in the marine administrative integrated DEMATEL with ANP. This will significantly improve the maritime relations personnel’s perception of maritime safety, as well as how the functional dimension benefit from each other and the accidents occurrence. After the completion of the questionnaire, 30 questionnaires were conducted. The balanced scorecard is a strategic planning and management system that is used extensively in many scopes; the maritime industry also needs a balanced scorecard concept to manage or process maritime accidents.

The empirical results are applied to provide the necessary factors to develop and improve a risk measured standard, which could be a reference for the shipping industry. From the empirical results, five criteria or key factors are determined to strengthen the risk assessment of marine accidents. Regarding the seafarer professional ability factor, shipping companies can use multi-standard methods to develop a conceptual framework for evaluating seafarers’ ability; regarding the ability to respond to emergencies factor, it is necessary to train some crew members to have a new safety concept of the new voyage instrument; Regarding the seafarer satisfaction factor, job satisfaction is considerably correlated with job performance of seafarers. In addition, the amount of stress associated with working onboard a ship and attractiveness of rewards are key determinants of job satisfaction. Regarding the accident information compilation factor, it is more important in the field of health and safety, especially in the maritime industry. Therefore, the shipping industry evaluates the importance of seafarers’ personality traits and workplace risk behavior. Regarding the seafarer education factor, the strengthening and upgrading of maritime education is the main link to coordinate with the national economic construction.

Finally, this paper illustrated a designed strategy map to enhance risk accident analysis. Like the BSC dimension, internal processes promote the customer satisfaction. The strategy map aims at the financial results of the enterprise, and the maritime safety report is just like the financial dimension of BSC. It is hoped that this paper can assist maritime authorities to reduce the occurrence of maritime accidents.

## Figures and Tables

**Table 1:** Maritime accident assessment factors.

Perspective			
Learning and Growth (A)	Internal Processes (B)	Seaworthiness (C)	Safety Report (D)
A1: Seafarer education	B1: Ability to respond to emergencies	C1: Voyage Seaworthiness	D1: Marine investigation
A2: Seafarer satisfaction	B2: Marine management efficiency enhancement	C2: Voyage equality	D2: Safety report
A3: Seafarer professional ability	B3: Training in environmental hygiene and cleaning operation	C3: Market share	D3: Recurrence rate of maritime accidents
A4: Seafarer resignation rate	B4: Accident information compilation	C4: Cargo owner relationship management	D4: Subsequent handling of maritime accidents
A5: Seafarer knowledge sharing			

**Table 2:** Total-influence matrix matrix *T*: four perspectives.

Perspective	A	B	C	D	Row Sum (d_i_)	Column Sum (r_i_)	d_i_ + r_i_	d_i_ − r_i_
A	0.54	1.027	1.016	1.138	3.721	2.044	5.765	1.677
B	0.701	0.648	1.044	1.078	3.471	2.044	5.515	1.427
C	0.584	0.739	0.65	1.065	3.038	2.044	5.082	0.994
D	0.219	0.311	0.419	0.322	1.271	2.044	3.315	−0.773

Footnote: A = learning and growth, B = internal processes, C = seaworthiness, D = safety report.

**Table 3:** Sum of influences given and received on criteria.

Perspective/Criteria	Row Sum (d_i_)	Column Sum (r_i_)	d_i_ + r_i_	d_i_ − r_i_
A. Learning and growth	3.721	2.044	5.765	1.677
A1. Seafarer education	2.019	2.339	4.358	−0.32
A2. Seafarer satisfaction	2.792	1.459	4.251	1.333
A3. Seafarer professional ability	2.387	2.192	4.579	0.195
A4. Seafarer resignation rate	1.653	2.206	3.859	−0.553
A5. Seafarer knowledge sharing	1.852	1.306	3.158	0.546
B. Internal processes	3.471	2.044	5.515	1.427
B1. Ability to respond to emergencies	2.849	2.538	5.387	0.311
B2. Marine management efficiency enhancement	2.192	2.634	4.826	−0.442
B3. Training in environmental hygiene and cleaning operation	1.987	1.211	3.198	0.776
B4. Accident information compilation	3.519	1.757	5.276	1.762
C. Seaworthiness	3.038	2.044	5.082	0.994
C1. Voyage Seaworthiness	2.391	1.904	4.295	0.487
C2. Voyage equality	2.159	2.364	4.523	−0.205
C3. Market share	1.329	2.658	3.987	−1.329
C4. Cargo owner relationship management	1.236	2.61	3.846	−1.374
D. Safety report	1.271	2.044	3.315	−0.773
D1. Marine investigation	1.203	1.283	2.486	−0.08
D2. Safety report	1.194	1.204	2.398	−0.01
D3. Recurrence rate of maritime accidents	1.389	1.152	2.541	0.237
D4. Subsequent handling of maritime accidents	1.853	1.666	3.519	0.187

**Table 4:** Weights and ranking for the maritime accident risk factors.

Perspective/Criteria	Local Weights	Global Weights	Ranks
*A. Learning and growth*	0.357	-	1
A1. Seafarer education	0.206	0.074	5
A2. Seafarer satisfaction	0.215	0.077	3
A3. Seafarer professional ability	0.243	0.087	1
A4. Seafarer resignation rate	0.191	0.068	7
A5. Seafarer knowledge sharing	0.145	0.052	13
*B. Internal processes*	0.283	-	2
B1. Ability to respond to emergencies	0.275	0.078	2
B2. Marine management efficiency enhancement	0.243	0.069	6
B3. Training in environmental hygiene and cleaning operation	0.215	0.061	9
B4. Accident information compilation	0.267	0.076	4
*C. Seaworthiness*	0.236	-	3
C1. Voyage Seaworthiness	0.239	0.056	11
C2. Voyage equality	0.232	0.055	12
C3. Market share	0.273	0.064	8
C4. Cargo owner relationship management	0.256	0.060	10
*D. Safety report*	0.124	-	4
D1. Marine investigation	0.236	0.029	16
D2. Safety report	0.233	0.029	17
D3. Recurrence rate of maritime accidents	0.242	0.030	15
D4. Subsequent handling of maritime accidents	0.289	0.036	14

## Data Availability

Not applicable.
